# Primary Osteoporosis Induced by Androgen and Estrogen Deficiency: The Molecular and Cellular Perspective on Pathophysiological Mechanisms and Treatments

**DOI:** 10.3390/ijms252212139

**Published:** 2024-11-12

**Authors:** Shao-Heng Hsu, Li-Ru Chen, Kuo-Hu Chen

**Affiliations:** 1 Department of Medical Education, Taipei Tzu-Chi Hospital, The Buddhist Tzu-Chi Medical Foundation, New Taipei City 231, Taiwan; hsupeter66900321@gmail.com; 2 Department of Physical Medicine and Rehabilitation, Mackay Memorial Hospital, Taipei 104, Taiwan; gracealex168@gmail.com; 3 Department of Mechanical Engineering, National Yang Ming Chiao Tung University, Hsinchu 300, Taiwan; 4 Department of Obstetrics and Gynecology, Taipei Tzu-Chi Hospital, The Buddhist Tzu-Chi Medical Foundation, New Taipei City 231, Taiwan; 5 School of Medicine, Tzu-Chi University, Hualien 970, Taiwan

**Keywords:** osteoporosis, estrogen, androgen, osteoprotegerin, Wnt signaling pathway

## Abstract

Primary osteoporosis is closely linked to hormone deficiency, which disrupts the balance of bone remodeling. It affects postmenopausal women but also significantly impacts older men. Estrogen can promote the production of osteoprotegerin, a decoy receptor for RANKL, thereby preventing RANKL from activating osteoclasts. Furthermore, estrogen promotes osteoblast survival and function via activation of the Wnt signaling pathway. Likewise, androgens play a critical role in bone metabolism, primarily through their conversion to estrogen in men. Estrogen deficiency accelerates bone resorption through a rise in pro-inflammatory cytokines (IL-1, IL-6, TNF-α) and RANKL, which promote osteoclastogenesis. In the classic genomic pathway, estrogen binds to estrogen receptors in the cytoplasm, forming a complex that migrates to the nucleus and binds to estrogen response elements on DNA, regulating gene transcription. Androgens can be defined as high-affinity ligands for the androgen receptor; their combination can serve as a ligand-inducible transcription factor. Hormone replacement therapy has shown promise but comes with associated risks and side effects. In contrast, the non-genomic pathway involves rapid signaling cascades initiated at the cell membrane, influencing cellular functions without directly altering gene expression. Therefore, the ligand-independent actions and rapid signaling pathways of estrogen and androgen receptors can be harnessed to develop new drugs that provide bone protection without the side effects of traditional hormone therapies. To manage primary osteoporosis, other pharmacological treatments (bisphosphonates, teriparatide, RANKL inhibitors, sclerostin inhibitors, SERMs, and calcitonin salmon) can ameliorate osteoporosis and improve BMD via actions on different pathways. Non-pharmacological treatments include nutritional support and exercise, as well as the dietary intake of antioxidants and natural products. The current study reviews the processes of bone remodeling, hormone actions, hormone receptor status, and therapeutic targets of primary osteoporosis. However, many detailed cellular and molecular mechanisms underlying primary osteoporosis seem complicated and unexplored and warrant further investigation.

## 1. Introduction

With an escalating global population of elderly individuals, osteoporosis, significantly associated with the likelihood of fragility fractures that lead to various patient complications, is quickly emerging as one of the most prevalent medical conditions, with estimates that it will affect 500 million individuals worldwide by 2025 [1]. Moreover, osteoporosis is often referred to as a ‘silent disease’ because it usually does not produce noticeable symptoms until it leads to fractures. According to the statistics presented by The International Osteoporosis Foundation (IOF), up to half of all women and one in five men aged 50 or older are likely to experience a fragility fracture during their lifetime [2]. Osteoporosis is often viewed as a disease predominantly affecting women, and this perception is largely due to the substantial increase in its incidence and fracture risk among women post-menopause as a result of hormonal changes. It is indisputable that women have a higher tendency to develop osteoporotic fractures than men [3]. Although men experience fractures less frequently than women, they face higher mortality rates following a fracture [4]. Hence, we should never neglect the importance of deficiencies in both androgen and estrogen, and how they affect bone metabolism in both genders respectively and collectively.

Osteoporosis is defined by a decrease in bone density, which gives rise to microarchitecture deterioration, and constitutes both significant public health and economic issues; it is characterized by its high occurrence rate and the severe clinical outcomes associated with it. According to a report regarding the burden and management of fragility fractures completed by Borgström et al. in 2020 that compared fragility fracture-related DALYs (disability-adjusted life years) with 16 other prevalent non-communicable diseases across six European countries, osteoporotic fractures ranked as the fourth most burdensome condition, exceeded only by ischemic heart disease, dementia, and lung cancer [5]. According to one 2021 meta-analysis review by Nader et al., the prevalence of worldwide osteoporosis was reported to be 18.3%. Under the different pooling sample sizes, women accounted for 23.1% while men constituted 11.7% in the general population, respectively [6]. Also, the highest prevalence of osteoporosis in the eldery was reported to be 24.3% in Asia [6]. A nationwide population-based study in Taiwan by Lee et al., utilizing data from Taiwan’s National Health Insurance Research Database from 2008 to 2019, found that the age-standardized incidence rates were 640 and 1372.5 per 100,000 people, respectively [7]. In the United States, osteoporosis leads to around 1.5 million fractures annually, with most of these incidents occurring in women who have gone through menopause [8]. A 50-year-old white woman’s lifetime risk for a hip fracture is estimated at 15–20%, with a 50% chance of incurring any osteoporotic fracture [8,9]. Frailty fractures due to osteoporosis primarily occur in the hip and spine, but can also be found in the proximal humerus, wrist, ribs, ischiopubic branches, and even in the ankle [10]. Among these fractures, vertebral and hip fractures are associated with an increased risk of death [11]. Moreover, women aged 50 and older experience osteoporosis at a rate four times greater and osteopenia at a rate twice as high as men, while men tend to have a greater risk of mortality after a hip fracture [12].

Therefore, with today’s increase in life expectancy, enhanced awareness regarding the prevention and recognition of osteoporosis among physicians and patients is beneficial for public health. Certain risk factors of osteoporosis-related fractures should be identified, such as aging, sex steroid hormone deficiency, low body mass, prolonged immobility, parental history of hip fracture, smoking and alcohol habits, chronic use of glucocorticoids, and vitamin D insufficiency [13]. The modifiable and non-modifiable risk factors of osteoporosis are listed in Table 1.

In clinical practice, osteoporosis can be classified into primary and secondary causes. Primary osteoporosis, a kind of osteoporosis that develops independently of other health conditions, is often related to aging and the decreased sex hormone levels that occur after menopause in women and andropause in men.

Primary osteoporosis can be further classified into type 1, type 2, and idiopathic types. Type 1 involutional osteoporosis, also referred to as postmenopausal osteoporosis, is a condition resulting from estrogen deficiency, and mainly impacts the spongy, trabecular bone [13]. Although this condition affects both men and women, it predominantly occurs in women aged 51 to 75, who are characterized by accelerated bone loss during this period [14]. Type 2 involutional osteoporosis, also referred to as senile osteoporosis, typically affects individuals aged 75 and older. As a result of the aging process, this condition leads to the degeneration of both the trabecular and cortical bone [13]. Idiopathic osteoporosis is a heterogenous type affecting both sexes in young adults. It involves compromised bone structure and strength, which is largely due to reduced osteoblast activity and inadequate bone formation during growth phases [15].

On the other hand, secondary osteoporosis results from numerous medical conditions or treatments deemed to be external factors that disrupt the bone turnover processes and negatively affect bone density and strength. Potential causes include prolonged use of glucocorticoids, thyroid dysfunction, or chronic diseases affecting nutrient absorption or hormone levels, and others [16]. Table 2 summarizes the possible medical conditions or treatments that can induce secondary osteoporosis.

The underlying molecular basis of osteoporosis is commonly attributed to enhanced osteoclast function and reduced osteoblast activity, or both. This imbalance in bone remodeling leads to increased bone loss and decreased bone production. In this review, we are going to discuss and explore the comprehensive pathophysiology of primary osteoporosis from the molecular and cellular point of view. Additionally, the effects of androgen and estrogen on bone metabolism will be discussed in this study.

## 2. Bibliographic Strategy of Literature Review

In this review, the literature was searched for basic and clinical studies that investigated the underlying molecular and cellular mechanisms of osteoporosis, related pathophysiological changes, and hormonal effects, as well as its treatment, following the process of database searching, screening, and inclusion of the references. In the first stage, all of the studies were collected from the databases Ovid Medline and PubMed using the search terms “osteoporosis”, “estrogen”, “androgen”, and “Wnt signaling pathway” for the research topic. For screening and selection in the following stage, only full-text articles were considered for inclusion for further analysis. In the second stage, duplicated articles were also excluded. Subsequently, two experts in the field independently inspected the contents of articles, focusing on such criteria as research materials, study designs, and results and/or outcomes, to identify eligible studies for further inclusion. Articles collected with questionable research methods, poor study designs, or mismatched results/outcomes were excluded at this stage. Any discrepancies between both experts were discussed by mutual communication to reach a consensus. All eligible articles were included in this review using the search terms and strategies described above. Finally, a total of 207 articles were selected for the current review.

The strength of the review lies in its extensive collection of all eligible articles in the literature to ensure sufficient and novel content in the field of osteoporosis. On the other hand, a limitation of this review is in the selection and exclusion of the articles by the subjective judgement of scholars, which may inevitably lead to selection bias.

## 3. The Commonly Used Diagnostic Tools, Diagnostic Criteria and Clinical Presentations for Osteoporosis

### 3.1. Common Diagnostic Tools

The diagnosis of osteoporosis relies on diagnostic tools that evaluate bone mineral density (BMD) and bone fragility through various methods. For checking BMD, these tools include dual-energy X-ray absorptiometry (DEXA), single photon absorptiometry, quantitative computed tomography (QCT), quantitative ultrasound (QUS), and other imaging techniques like digital X-ray radiogrammetry, radiographic absorptiometry, ultrasonography, and magnetic resonance imaging [17,18,19,20]. In terms of fracture risk evaluation, the Garvan fracture risk calculator, QFracture^®^, and FRAX^®^ are available tools used to estimate the risk of fractures, each employing different parameters and algorithms to evaluate an individual’s likelihood of experiencing a bone fracture [21,22].

### 3.2. The Diagnostic Criteria and Clinical Presentations

In diagnosing osteoporosis, it is fundamental to assess the BMD of the femoral neck and lumbar spine (L1–L4) using DXA, which is considered the gold standard for measuring BMD due to its extensive validation and global acceptance in clinical practice [23].

According to the guidelines from the International Osteoporosis Foundation (IOF), osteoporosis diagnostic criteria are based on T-score thresholds established by the World Health Organization (WHO), these criteria categorize bone density and are listed as follows [24]:

(1) Normal: A T-score of −1.0 or above indicates a bone density within the normal range.
(2) Osteopenia (Low Bone Mass): A T-score between −1.0 and −2.5 suggests low bone mass, or osteopenia, which is a precursor to osteoporosis.
(3) Osteoporosis: A T-score of −2.5 or lower signifies osteoporosis, indicating significant bone loss and increased fracture risk.
(4) Severe (Established) Osteoporosis: A T-score of −2.5 or lower, accompanied by one or more fragility fractures, is categorized as severe osteoporosis, emphasizing a heightened risk of future fractures and severe bone deterioration.

With regard to clinical presentation and physical examination findings, patients are encountered with the following:

(1) Fragility fractures: these fractures occur from minimal trauma, such as falls from standing height, and are most common in the hip, spine, and wrist.
(2) Back pain: spinal fractures may cause acute or chronic back pain, which is sometimes the first noticeable sign.
(3) Height loss: progressive vertebral fractures may result in a gradual reduction in height.
(4) Deformities: the curvature of the upper spine is often seen in advanced cases of kyphosis, giving a rounded-back appearance, which is called Dowager’s Hump [25].
(5) Decreased motility: spinal deformities or fractures can reduce the range of motion in the spine and impact overall mobility.
(6) Tenderness over spine: point tenderness along the spine may indicate recent or ongoing vertebral fractures [26].
(7) Balance and muscle weakness: associated muscle weakness, especially in the back and lower limbs, can lead to balance issues and an increased fall risk.

## 4. The Structure Biology of Bone

The human skeleton, which is structurally complex and diverse, evolves from 270 bones in infancy to 206 in adulthood due to bone fusion during growth. It consists of the axial skeleton (skull, spine, sternum, ribs) and appendicular skeleton (femur, radius), and serves vital functions such as organ protection, movement facilitation, and muscle leverage. Besides structural support, it is metabolically active, regulating mineral homeostasis, enabling hematopoiesis in bone marrow, and storing minerals like calcium, phosphate, and carbonate, all structured around an extracellular matrix and osteocytes [27].

At a macroscopic level, bone is primarily composed of two distinct types of structures: cortical (compact) and trabecular (spongy). Cortical bone, forming about 80% of the skeleton, creates a compact osseous shell with osteons. It is nourished by Haversian and Volkmann’s canals as well as canaliculi [28,29], and is enveloped by connective tissues known as the periosteum and endosteum. Cortical bone has a lower surface-to-volume ratio compared to trabecular bone. With age or illness, cortical bone becomes more porous, increasing its surface area while reducing its strength. This increased porosity, especially near the periosteal surface of long bones, leads to structural changes [30] and makes the elderly more predisposed to fractures.

In terms of trabecular bone, it has a higher surface-to-volume ratio, and constitutes 20% of total mineralized bone—including the ends of long bones and flat bones, which undergo rapid remodeling and bone loss—but is metabolically four times more active than cortical bone. Although the skeleton contains four times more cortical bone than trabecular bone, their overall impacts on bone turnover are equivalent due to the estimation that bone turnover occurs at a rate four times higher in trabecular bone compared to cortical bone [31].

Bone is a mineralized tissue comprised mainly of 60% inorganic substances, predominantly hydroxyapatite to provide the bone with strength, stiffness, and resistance to compressive forces, 30% organic materials like proteins, and 10% water [32]. The organic bone matrix, serving as a resistance cushion to tensile force, contains five types of collagen—mainly type 1, which renders the triple helix structure to contribute to bone’s structural integrity and mechanical strength—and non-collagenous proteins, such as osteocalcin, osteonectin, osteopontin, bone sialoprotein, and RANKL. These proteins play important roles in various biological processes, including mineralization, bone remodeling, osteoid formation, cell signaling, and regulation of bone cell activity [27,32].

Water contributes significantly to bone tissue’s composition, residing in the collagen matrix to aid in keeping the bone hydrated and flexible. Additionally, the water distribution is thought to be present in three forms: bulk or free water, which is unattached; bound water, which is attached to other molecules; and structural water, which is an integral part of the matrix’s structure [33]. Free water in bone can circulate through the pores, vascular channels, and central (Haversian and Volkmann) canals, as well as within the lacunar-canalicular system in order to carry metabolic nutrients and wastes. Loosely bound water facilitates load transfer between collagen and minerals, enabling movement at their junctions. This process lessens shear stress, enhancing the overall toughness of the tissue [34,35]. With the absence of bound water, the dehydrated state of the bone makes the tissues more brittle, which has the most dominant impact on bone strength and toughness [35]. The structural water, trapped within the imperfected carbonated apatite crystal lattice of bones, provides structural stability by creating hydrogen bonds that link adjacent ions [36]. Moreover, structural water is believed to act as a medium that helps mineral platelets and hydroxyapatite layers within the collagen matrix of the bone to stay organized, facilitating the formation of a continuous cross-fibrillar phase amidst the irregular collagen fibrils [37].

## 5. The Principal Cells Involved in Bone

There are five primary types of bone cells which can be identified in the bone tissue.

### 5.1. Osteoprogenitor Cells (OPCs)

Bone repair and regeneration are initiated by osteoprogenitor (osteogenic) cells found in various locations, such as the epiphysis, marrow [38], growth plate, cranial suture [39], bone canals, periosteum, and endosteum [40]. These cells have the potential to differentiate into osteoblasts. However, their ability to proliferate and differentiate diminishes with age, especially after menopause, affecting the bone’s capacity to regenerate and repair [41].

The manifestations of OPCs can be affected by the interaction of sex hormones and their corresponding receptors. Moreover, Pirro et al. have demonstrated the significance of osteoprogenitor cells in osteoporosis by identifying a markedly higher count of AP+/CD34+ and OCN+ cells in osteoporotic postmenopausal women than those in healthy controls. Furthermore, the presence of CD34+/OCN+ cells has been correlated with decreased BMD, highlighting their potential role in the pathophysiology of the disease [42].

### 5.2. Osteoblasts (OBLs)

Osteoblast differentiation from pluripotent mesenchymal progenitors relies on the orderly expression of the two critical transcription factors RUNX2 and OSX1 [43]. These osteoblasts are instrumental in bone formation in the developing skeleton and during the process of bone remodeling. They also create packed layers on the bone’s surface, project their processes into the forming bone and synthesize various molecules, such as enzymes, growth factors, and hormones—including alkaline phosphatase, collagenase, TGF-β, IGFs, osteocalcin, and type 1 collagen—to facilitate new bone formation [44]. In contrast, the secretion of binding proteins by osteoclasts, such as osteocalcin and osteonectin, helps regulate the deposition of minerals inside the bone tissue [27].

The manifestations of OBLs can also be affected by the interaction of sex hormones and other corresponding receptors. The creation of mouse models with specific deletions of the estrogen receptor (ER) or the androgen receptor (AR) at various stages of osteoblast differentiation has provided significant insights into the effects of sexual hormones [45]. By targeting different points in the osteoblast lineage, from pluripotent progenitors to mature osteoblasts and osteocytes, researchers have been able to study the direct effects of estrogen and androgen signaling on bone cell development and function [46,47]. For example, using genetic tools like Prx1-Cre (paired related homeobox 1-Cre recombinase) and Osx1-Cre to remove ERs from early osteoblast progenitors diminishes cortical bone mass and periosteal bone formation, illustrating the crucial role of these receptors in bone growth and maintenance [46].

### 5.3. Osteoclasts (OCLs)

Derived from hematopoietic stem cells, osteoclasts are large, multinucleated cells vital for bone breakdown [48]. Their significant metabolic activity facilitates the efficient resorption of bone tissue. Because of the key feature of a ruffled border within the osteoclast cytoplasm, a complex folded membrane has the ability to form extensive microvilli-like extensions that press against the bone surface where bone resorption occurs.

Moreover, with the secretion of hydrogen ions, tartrate-resistant acid phosphatase (TRAP), and cathepsin K enzymes, osteoclasts acidify the bone surface beneath the cell; TRAP is involved in the degradation of the bone matrix protein, and cathepsin K breaks down the proteinaceous matrix, mostly type 1 collagen, respectively [49,50]. In terms of osteoclastogenesis, osteoclast formations are driven by two essential cytokines, RANKL (receptor activator of nuclear factor kappa-B ligand) and M-CSF (macrophage colony-stimulating factor), both of which differentiate macrophage colony-forming units into mononuclear macrophages [18,48], which are then further assembled into multinucleated osteoclasts with a number of nuclei ranging from two to eight [46].

The manifestations of OCLs can also be affected by the interaction of sex hormones and their corresponding receptors. When it comes to the effects of estrogen and androgen on osteoclasts, mouse models receiving the targeted deletion of specific receptors like ERα, AR, or RANKL, and the use of selective estrogenic compounds, have revealed a better understanding of the roles and signaling pathways of ERα, AR, and estrogens in osteoblasts [45]. For instance, removing the *Esr1* gene from mature osteoclasts in female mice increases their number in trabecular bone and decreases bone density, due to a longer osteoclast lifespan that is linked to lower Fas ligand levels. However, ERα deletion in mature osteoclasts does not affect bone density in male mice [51]. Based on these findings, researchers have summarized that in females, estrogen contributes to bone preservation by enhancing Fas ligand expression, which in turn facilitates the apoptosis of osteoclasts.

Similarly, research by Määttä et al., using mice with AR specifically removed from osteoclasts, has shown that the action of androgens in these bone-resorbing cells does not contribute to androgens’ protective effects on either the trabecular or cortical bone structures [52]. Hence, estrogen and its receptors predominantly regulate osteoclast activity, while testosterone influences osteoclasts indirectly by converting to estrogen (estradiol; E2) or by suppressing interleukin-6 (IL-6) production, which is necessary for osteoclast precursor maturation [53].

### 5.4. Osteocyte (OS)

Osteocytes are considered terminally differentiated bone cells arising from osteoblasts, which are distributed all over the entire skeleton. They have a long lifespan of up to 50 years, outnumbering osteoclasts by a thousandfold and osteoblasts by tenfold [54]. The Wnt signaling pathway in osteocytes is crucial for detecting mechanical strain (mechanosensing) and regulating bone mass [55]. Osteocytes use the Wnt signaling pathway to sense changes in mechanical load and respond to the conditions by adjusting bone formation and resorption, thereby maintaining bone strength and health [56]. However, sclerostin, a unique protein that blocks the Wnt signaling pathway, seems to play a crucial role in the bone’s response to external mechanical stress, as well as facilitates bone resorption [57]. Furthermore, sclerostin has shown an antagonistic effect on the co-receptor signaling of Lrp5 (low-density lipoprotein receptor-related protein 5) as well. Overall, the regulation of sclerostin in osteocytes is a precise mechanism that orchestrates bone growth both regionally and locally in reaction to increased mechanical stress, possibly by modifying the pathway of the Wnt/Lrp5 signaling [58].

The manifestations of OS are also affected by the interaction of sex hormones and their corresponding receptors. ERα, ERβ, and GPER1 (GPCR30) are all expressed in osteoblasts, osteocytes, and osteoclasts, indicating their significant roles in bone cell function and regulation [59,60]. Nonetheless, these molecular and cellular expressions have different patterns. For example, the distribution of ERα in osteocytes is more prevalent in cortical bone than that in trabecular bone [59], while ERβ in osteocytes shows higher expression in trabecular bone [59]. The absence of GPER1 leads to reduced bone growth in female mice [61], but paradoxically results in increased bone mass in male mice [62].

### 5.5. Bone Lining Cells (BLCs)

Some osteoblasts remain flattened out and cover the surfaces of the inactive bone, becoming less specialized and therefore referred to as lining cells [54]. Although BLCs are dormant on the surface of the bone, they can be reactivated to become bone-forming cells again after being activated by particular signals.

Additionally, BLCs appear to have a remarkable role in bridging the process of bone resorption with bone formation [63]. These cells can also act as intermediaries, preventing osteoclasts from directly contacting the bone matrix to inhibit bone resorption when unnecessary [64]. Furthermore, they contribute to osteoclast differentiation as well by producing both OPG and RANKL, which positively and negatively regulate bone remodeling processes [64,65].

The manifestations of BLCs can be affected by the interaction of sex hormones and their corresponding receptors. Several experiments conducted by Carmen et al., using techniques such as immunohistochemistry, mRNA analysis by qRT-PCR, and the novel technology of laser capture microdissection (LCM), have revealed that estrogen significantly influences the expression of RANKL in bone lining cells (BLCs) within living organisms. Moreover, estradiol (E2), the most potent estrogen, effectively reduces RANKL expression in BLCs. Generally, RANKL binds to the RANK receptor on osteoclasts and their precursors to promote bone resorption. In contrast, osteoprotegerin (OPG) acts as a decoy by binding to RANKL, rendering it inactive and preventing bone loss. When estrogen levels fall, the suppression of RANKL diminishes, leading to increased bone resorption by osteoclasts. Furthermore, estradiol is thought to influence the production of OPG in bone lining cells [66].

## 6. Bone Remodeling

Before analyzing the roles of estrogen and androgen in bone metabolism, it is necessary to understand the process of bone remodeling, which is an essential process for maintaining mechanical strength that is influenced by various factors, such as hormonal signals, paracrine and autocrine factors, and the mechanical stress exerted by physical load [19]. The key signaling pathways of bone remodeling are primarily governed by the RANK/RANK ligand/osteoprotegerin (OPG) and canonical Wnt signaling pathways. Paracrine control of this process involves cytokines, growth factors, and prostaglandins, while the endocrine influences come from hormones such as the parathyroid hormone, vitamin D, calcitonin, growth hormone, glucocorticoids, sex hormones, and thyroid hormone [20].

**Figure 1:**
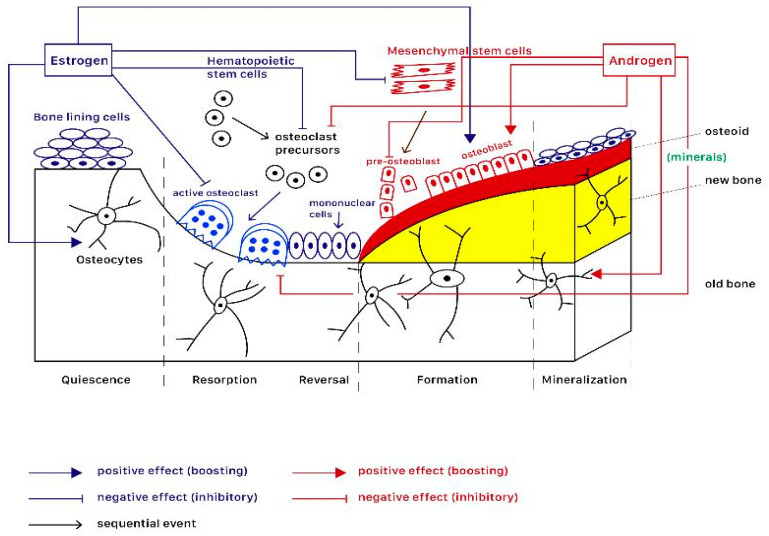
Both estrogens and androgens affect the formation and lifespan of osteoclasts and osteoblasts, as well as the longevity of osteocytes. Thick arrowheads and bookends in this figure respectively demonstrate the positive and negative impacts of sex steroids on the formation and survival of these cells.

Figure 1 illustrates an organized and hierarchical sequence in distinct phases of bone remodeling [18]. Five distinct phases complete the cycle of bone remodeling, which consists of quiescence, resorption, reversal, formation, and mineralization. Bone remodeling initiates in the quiescence stage, in which bone surface lies dormant before signaling recruits pre-osteoclasts and bone surface exposure by separating bone lining cells, leading to the resorption stage, in which osteoclasts dismantle old bone. The process then moves to the reversal phase after the bone is resorbed, in which the surface is primed for new bone matrix deposition, and signaling processes then ensure the coordination of bone resorption with formation. Finally, osteoblasts first create a collagen-rich layer called osteoid, then help in turning this layer into hard bone. The cycle concludes with the mineralization stage, in which the new bone matrix solidifies and matures.

## 7. Androgens and Estrogens: Their Roles in Bone Health and Osteoporosis

Testosterone (T) is a vital male sex hormone predominantly produced in the testicles, although it can also be found in the adrenal glands. It governs several fundamental bodily functions, impacting sexual development, reproductive health, and secondary sexual characteristics, such as muscle strength, bone density, and body hair growth. As a C19 steroid, it is produced from cholesterol and associated derivatives, in a way similar to adrenal androgens (A). T from both gonadal and adrenal sources can be transformed into C18 steroids, known as 7-beta estradiol (E2), by the enzyme P450 aromatase (B). Encoded by the CYP 19A1 gene, this enzyme is present in several peripheral tissues, especially in bone and adipose tissues [67].

**Figure 2:**
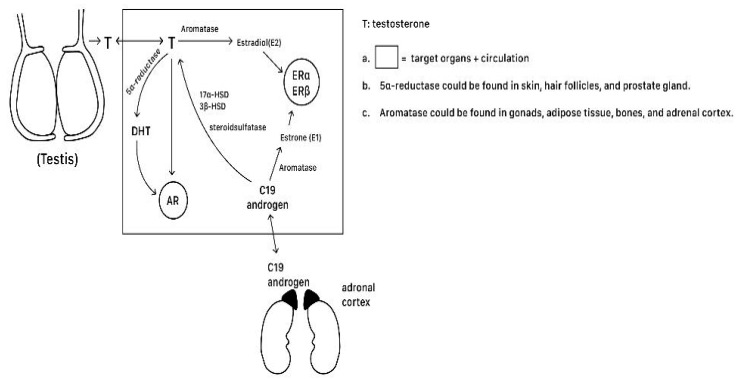
The production and action of testosterone on bone remodeling. Testosterone can act as an enhancer of bones via signal transduction that includes its direct binding to AR and indirect binding to ER via the subsequent production of estradiol from testosterone. DHT: dihydrotestosterone; AR: androgen receptor; ER: estrogen receptor; HSD: hydroxysteroid dehydrogenase.

Testosterone significantly regulates bone modeling and remodeling by promoting osteoblast differentiation and proliferation while inhibiting osteoclast maturation and activity. Exerting anabolic effects on bone tissues, testosterone can act as an enhancer of bones via signal transduction, which includes its direct binding to AR and indirect binding to ER via the subsequent production of estradiol from testosterone (Figure 2). Also, testosterone is metabolized into dihydrotestosterone (DHT) by local enzyme 5-reductase (SRD5A; type I and II) in target tissues like skin, hair follicles, and the prostate gland [68]. DHT, the strongest form of androgen, has a twofold higher affinity and a fivefold decreased dissociation rate compared to testosterone, and it directly interacts with the androgen receptor (AR) to initiate multiple biological actions [46,69].

Sex hormones serve as critical contributors to the development of skeletal structures in three dimensions, which includes longitudinal and radial bone growth. Androgens are believed to promote longitudinal bone growth through their direct impact on growth plate chondrocytes, as demonstrated strictly in vivo [70]. Additionally, a cross-sectional study by S. Vandewalle et al. [71] revealed that higher levels of testosterone govern periosteal apposition and enhance bone growth, with the result that males typically have larger periosteal circumference than females [72]. On the other hand, E2 restricts the expansion of periosteal bone while enhancing the apposition of endosteal bone in females [73].

With regard to puberty, sex hormones have been proven to present biphasic effects combined with exemplified interactions of growth hormones on bone growth [72,74]. During the onset of puberty, relatively low levels of estrogens and androgens trigger a growth spurt by elevating levels of growth hormone (GH) and insulin-like growth factor (IGF-I) [46]. This surge is primarily stimulated by estrogens, which likely exert their stimulatory effects via indirect actions on GH and IGF-1 [75]. Additionally, estrogens display biphasic effects on epiphyseal growth. In lower concentrations, typically physiologically found in males, estrogens stimulate bone growth, while in higher concentrations, common in females, estrogens are associated with the cessation of bone growth [75,76]. This dual action of estrogen explains its different impacts on skeletal development across genders.

Men’s bones are wider because they experience more periosteal expansion during puberty compared to women [76]. This kind of growth is influenced by the AR later in puberty and the ER earlier in puberty, but it is primarily driven by IGF-I, likely through the central conversion of androgens into estrogens [77]. Conditions featuring a deficiency in androgens, such as male hypogonadism, androgen-insensitive syndromes, and prostate cancer treated with androgen deprivation therapy, are closely linked to significant bone loss and a heightened risk of fractures. From a physiological point of view, total testosterone levels typically experience a slight decline with age; yet, for most elderly men, these levels stay above the critical threshold (200–300 ng/dL or 8–11 nM/L) that distinguishes normal functioning from symptomatic hypogonadism [78,79].

It is widely accepted that estrogen primarily affects bone by attenuating bone resorption, thereby controlling the bone remodeling process [51]. Even in adult men, an experimental study designed by Falahati-Nini et al. has shown statistically significant findings indicating that estrogens were responsible for at least 70% of the impact of sex steroids on bone resorption in these older men, whereas testosterone contributed to no more than 30% of this effect in terms of bone resorption markers [80]. Furthermore, the Swedish cohort from the Osteoporotic Fractures in Men Study revealed a pattern, in which it was not low levels of free testosterone but rather low levels of free estradiol that were significantly linked to increased fracture risk in older men (n = 2639, with an average follow-up of 3.3 years) [81]. In conclusion, men do not necessarily suffer from true adrenopause (regardless of testosterone level) in terms of osteoporosis. Instead, their total E2 concentrations should stay above the threshold to preserve bone health [70].

## 8. The Impacts of Hormone Deficiency on Osteoporosis Onset

The primary causes of osteoporosis include factors related to the natural aging process and increased bone resorption due to a deficiency in sex hormones. This bone resorption, particularly associated with E deficiency, affects both men and women, but it is more prevailing in women because of a significant drop in estrogen levels after menopause. In postmenopausal women, lower E2 levels cause circulating macrophages to produce pro-inflammatory cytokines such as IL-1, IL-4, IL-6, and TNF-α, which stimulate RANKL production, thereby activating osteoclasts and leading to greater trabecular bone loss. In contrast, in older men, a deficiency in E2 leads to lower BMD because of increased bone turnover, deteriorated microarchitecture, and accelerated bone loss [82]. With regards to testosterone levels in aging men, there is a reduction in total serum testosterone levels and an even more pronounced decline in free and bioavailable testosterone, partly due to a rise in sex hormone-binding globulin (SHBG) [83]. Likewise, men with hypogonadism not only have lower BMD but also exhibit poorer bone microarchitecture [84]. Recent reports by Maria et al. have further highlighted that hypogonadism is linked to deteriorated trabecular microarchitecture in the distal tibia [85], underscoring the extensive impact of this condition on bone structure and strength.

Interestingly, several studies have revealed the importance of testosterone replacement therapy (TRT) in men with hypogonadism. A controlled clinical trial conducted by Peter J. et al. showed that one year of TRT in older men significantly enhanced vertebral bone mineral density (vBMD) and estimated bone strength [86]. However, one study—a systematic review and meta-analysis by Zhichao et al.—showed that when compared with a placebo, TRT did not increase total BMD, cardiovascular events, all-cause mortality, or prostatic events. Instead, TRT was able to improve sexual function and quality of life [87]. These inconsistent results might be due to differences in sample sizes, duration, and dosage of TRT, all of which impact the robustness and comparability of the results.

**Figure 3:**
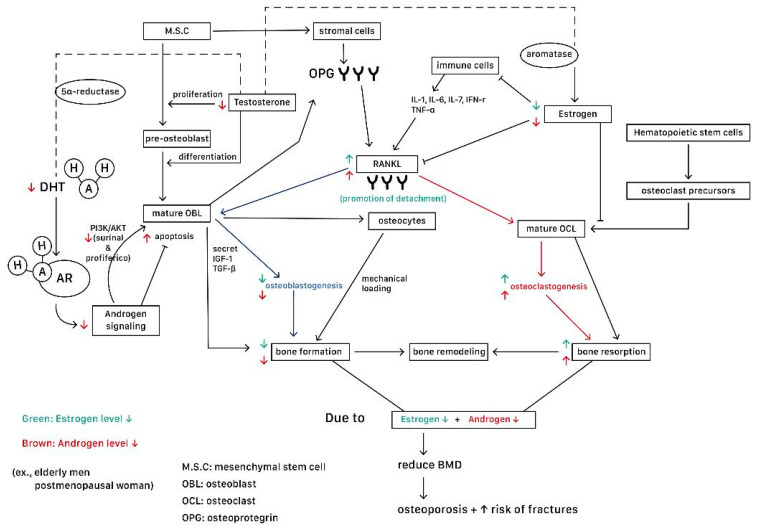
A summary of androgen and estrogen deficiencies in postmenopausal women and the elderly from a cellular and molecular point of view. DHT: dihydrotestosterone; BMD: bone mineral density; RANKL: receptor activator of nuclear factor kappa-B ligand; AR: androgen receptor.

Androgen deprivation therapy (ADT), which is used in treating prostate cancer patients, is also a major contributing factor to osteoporosis. The extensive application of ADT is linked to a high incidence of osteoporosis, affecting up to 53% of men diagnosed with prostate cancer [88]. Bone loss associated with ADT notably affects both trabecular and cortical bone, leading to a marked reduction in bone mineral density (BMD), particularly prominent in the first year of treatment. Moreover, ADT is definitively linked to a broader spectrum of issues, including muscle loss, deterioration of the bone microarchitecture, and a heightened risk of fractures [89,90]. One meta-analysis conducted by DoKyung Kim et al. demonstrated that in a group of men who had undergone ADT, there were statistically significant reductions in BMD compared to those in a control group. The reduction in BMD in the ADT group was categorized as follows: the lumbar spine (MD −3.60, 95% CI −6.72 to −0.47, *p* = 0.02), femoral neck (MD −3.11, 95% CI −4.73 to −1.48, *p* = 0.0002), and total hip (MD −1.59, 95% CI −2.99 to −0.19, *p* = 0.03). These results suggested a highly significant relationship between ADT and lower BMD in patients with prostate cancer [91]. Figure 3 illustrates a summary of androgen and estrogen deficiencies in postmenopausal women and the elderly from a cellular and molecular point of view. Also, special diseases which result in subsequent androgen or estrogen deficiencies and their impacts on BMD and bone health are listed in Table 3 and Table 4, respectively.

## 9. The Pathophysiology of Osteoporosis by Sexual Dimorphism

Gaining deeper insight into the gender-specific mechanisms of osteoporosis could lead to the creation of innovative, customized treatments and preventive strategies. This section summarizes the clinical manifestation of diseases linked to sex hormones and their impacts on bone caused by mainly primary hormone deficiencies and a few secondary hormone deficiencies, which are presented in Table 3 and Table 4.

## 10. The Androgen (A)/Androgen Receptor (AR) Signaling Mechanism of Action

Androgen can be defined as a high affinity ligand for AR. Also known as NR3C4 (nuclear receptor subfamily 3, group C, gene 4), the AR is part of the steroid hormone nuclear receptor (NR) family, whose members act as ligand-inducible transcription factors. Additionally, the NR family also includes the estrogen receptor (ER), glucocorticoid receptor (GR), progesterone receptor (PR), and mineralocorticoid receptor (MR) [92]. Similar to other members of the NR family, AR is composed of three main functional domains: the N-terminal domain (NTD), followed by the DNA binding domain (DBD), and concluding with the C-terminal ligand binding domain (LBD) [93]. In the absence of A, AR is found in the cytoplasm, where it is bound to heat shock proteins [93]. Acting from the cytoplasm to the nucleus, A binds to AR in the cytoplasm first, and then the forming A/AR complex is translocated by importin-α into the nucleus [93]. Within the nucleus, the AR part in the A/AR complex identifies and attaches to genomic areas that contain sequence motifs known as androgen response elements (AREs), functioning as a dimer. The majority of AR binding sites (ARBSs) are located in the promoter and enhancer regions of target genes [94].

Under the androgen receptor knockout male mice (ARKO) model, mechanically, Chawnshang Chang and Hong-Yo Kang et al. pinpointed several key genes that were essential for AR-mediated bone formation, including the tissue-nonspecific alkaline phosphatase (TNSALP)-encoded alkaline phosphatase 2 (Akp2) gene and the small integrin-binding ligand N-linked glycoprotein (SIBLING) gene family [95]. The A/AR complex can stimulate the expression of the TNSALP and SIBLING gene family by attaching to the ARE motifs located in their promoter region [96].

## 11. The Estrogen (E)/Estrogen Receptor (ER) Signaling Mechanism of Action

Estrogen is the principal hormonal regulator of bone metabolism in both women and men, and plays a vital role in maintaining bone balance, primarily through its interaction with estrogen receptors ERα, ERβ, and G-protein coupled estrogen receptor 1 (GPER1), which is also known as GPR30 and is responsible for the non-genotropic pathway. The former two receptors, which are responsible for the classic genomic pathway, can be found and expressed in numerous cell types, including osteocytes, osteoblasts, bone marrow mesenchymal stem cells (BM-MSCs), and osteoclasts.

In the classic genomic pathway, E penetrates the cell and attaches to the ERα and Erβ located in the cytoplasm. This complex then migrates to the nucleus, where it forms either homo- or heterodimers and directly binds to specific DNA sequences known as Estrogen Response Elements (EREs) [46]. Additionally, the second mechanism involves independent ERE: the E/ER complexes travel to the nucleus and engage in protein–protein interactions with other TFs, effectively sequestering them. This modifies their interactions with DNA, resulting in changes in gene expression [97].

In 2012, molecular cloning techniques led to the discovery of a new GPER [98], GPER1, which stemmed from the identification of the gene in 1997 [99]. E binding to GPER1 leads to interactions that trigger downstream signaling pathways that ultimately result in changes in gene expression. For instance, Xiaozong Lin et al. have demonstrated that GPR30 activation boosts the expression of Runx2, a crucial transcription factor for bone formation, and that GPR30 expression increases during osteoblast differentiation and substantially enhances key mineralization markers, such as alkaline phosphatase, osteocalcin, osterix, and type I collagen [100]. Recently, Chuang et al. (2020) found that GPER1/GPR30 facilitates the proliferation of bone marrow mesenchymal stem cells through the cAMP/PKA/CREB signaling pathway [101]. Estrogen inhibits the secretion of RANKL while promoting the release of osteoclast-inhibiting factors such as growth hormone, GLP-1, and OPG, thus reducing osteoclast activity. Conversely, a deficiency in estrogen results in increased apoptosis of osteoblasts and impedes their differentiation through various mechanisms.

## 12. Corepressors and Coactivators

The following mechanisms are transcriptional activities, which are modulated by AR coregulators, including coactivators that can amplify AR transactivation and corepressors that can inhibit AR transactivation [102].

Corepressors can perform the following actions: (1) inhibit AR’s DNA binding or nuclear translocation; (2) recruit histone deacetylases; (3) disrupt interactions between AR and its coactivators; (4) interfere with the connection between AR’s N-terminus and C-terminus; (5) serve as scaffolds for other AR coregulators; and (6) target the basal transcriptional machinery [102].

Among the corepressors, calreticulin is capable of binding to the amino acid sequence motif KXGFFKR present in the cytoplasmic domains of all integrin alpha-subunits. Particularly, the amino acid sequence KVFFKR, found in the DBD of AR, connects the attachment of AR with calreticulin, thereby inhibiting AR-DBD binding to its DNA response elements [103,104]. In a similar way, the corepressor FOXO1 directly engages with the N-terminal domain (NTD) of the AR to prevent its interactions with co-activators [105]. On the contrary, steroid receptor coactivator-1 (SRC-1) is a well-known coactivator for NRs [106]. Although SRC-1 has demonstrated effective interactions with AR and ERα, the majority of studies have focused on how AR-SRC-1 influences cell proliferation during prostate development and in prostate cancer [106,107,108]. On the other hand, research on the regulation of bone mineral density by ERα-SRC-1 has shown the significance of SRC-1 in protecting against osteopenia and osteoporosis regardless of sex [109,110,111]. Functioning as a coactivator, SRC-1 can maintain BMD, and its deficiency—for example, in SRC-1-knockout male and female mice—will result in trabecular bone loss. This finding is attributed to androgen resistance in males [109,112] and estrogen resistance in females [113].

Acting as another coactivator, FHL-2 is a protein encoded by the *FHL2* gene in humans that plays an important role in osteoblast differentiation [114]. Interestingly, in the nucleus, FHL2 serves as a co-activator for various transcription factors, such as CREB, AP-1, androgen receptor, and β-catenin. Given that these transcription factors and integrins are crucial for osteoblast function and bone formation [115], FHL2’s ability to enhance their activities could partially account for its osteogenic effects on bone health. In other words, the deficiency of the anabolic effect of FHL2 results in decreased activity of osteoblasts, subsequently leading to osteopenia and osteoporosis [116,117].

## 13. Ligand-Independent Pathways and Nongenomic Actions of Sex Steroids

Initially, it was assumed that androgen and estrogen could only impact transcription through their direct binding to AR to activate ARE or to ER to activate ERE, respectively. However, intriguing observations suggested that ERs could be activated in many cells even in the absence of estrogen or other receptor agonists [118], and that non-nuclear initiated actions were capable of affecting various physiological processes quickly, especially crucial in the field of GPER30 in bone tissues [100,119,120].

### 13.1. Ligand-Independent Signaling Pathways

The ligand-independent activation of ERs often occurs through the phosphorylation of specific residues, such as serine and tyrosine, or through their interaction with coregulators. This mechanism is facilitated by the regulatory molecules necessary for phosphorylation, including Protein Kinase A (PKA), Protein Kinase C (PKC), and components of the MAPK phosphorylation cascade, which target the receptors and modify their activity without the need for a traditional ligand [121,122]. ERα is especially crucial for the osteogenic response to mechanical loading in a ligand-independent manner, specifically through the actions of the AF-1 domain and not that of AF-2. To support this, evidence from a female mouse model indicates that the cortical loading response does not involve the classical genomic ERE-mediated pathways, suggesting a distinct mechanism of action [123].

### 13.2. Nongenomic Actions

ANGELS, standing for activators of nongenotropic estrogen–like signaling, are compounds or factors that initiate estrogen-like signaling through pathways that do not involve direct interactions with DNA. These pathways are typically known as non-genomic [97] because they do not involve changes in gene transcription initiated by the classic estrogen receptor pathway at the DNA level. Instead, ANGELS can activate cell signaling through membrane-bound or other related receptors in the cytoplasm, leading to rapid cellular responses [124] that do not involve nuclear localization of the ERs. This can involve various second messengers (Ca^2+^, IP3, and cAMP) and kinase pathways (PKC, ERK-MAPK, and PI3K-Akt), and affect cell functions like proliferation, migration, and survival without the genomic lag associated with transcription and translation [125,126]. Several studies have shown that ANGELS offer neuroprotective benefits, cardiovascular protection, and, especially, help prevent cortical bone loss—though not trabecular bone loss—following estrogen deprivation in female mice [127,128,129].

## 14. Management of Primary Osteoporosis

The management of primary osteoporosis can be classified into pharmacological treatment and non-pharmacological treatment. The pharmacological treatment can ameliorate osteoporosis and improve BMD via actions on different pathways. The non-pharmacological treatment includes nutritional support, exercise, as well as dietary intake of antioxidants and natural products. Aside from the diagnostic criteria and tools for osteoporosis we mentioned in the previous section, the IOF particularly emphasizes the importance of treatment adherence. For osteoporosis treatment to be effective, it must be taken as prescribed. However, many patients with osteoporosis face challenges in maintaining medication adherence. Adherence to oral bisphosphonates is notably low, with only 43% to 59% of patients continuing their medication after one year, and rates are even lower for generic versions, leading nearly half of all patients to discontinue treatment within the first year [130,131]. To detect or estimate drug adherence, the IOF and the International Federation of Clinical Chemistry (IFCC) proposed a screening strategy involving the measuring of biochemical markers of bone formation—serum procollagen type I N-terminal propeptide and bone resorption, serum collagen type I C-terminal telopeptide—after 3 months of oral bisphosphonate therapy [132]. These markers, which are accessible and affordable, offer early insights into a treatment’s effects on bone.

### 14.1. Pharmacological Treatment

The ultimate aim of pharmacological treatment is to minimize the risk of fractures. According to the guidelines of the American College of Physicians and with the latest recommendations and FDA approval on the pharmacological treatment of primary osteoporosis or low bone mass to prevent further fractures [16,133], pharmacological treatments can be grouped into six categories, including bisphosphonates, parathyroid hormone (PTH) analogs, RANK-ligand inhibitors, sclerostin inhibitors, estrogen-related therapy (estrogen supplement and selective estrogen receptor modulators), and calcitonin salmon. Except for estrogen-related therapy, which focuses on the female group, the other pharmacological treatments may apply to both genders in the treatment of osteoporosis. Specifically, males who are diagnosed with osteoporosis may benefit from androgen targeted therapy, which may exert its hormonal effects to treat osteoporosis by peripheral conversion. In clinical practice, the specific benefits and potential drawbacks of pharmacological treatments should be taken into consideration.

#### 14.1.1. Bisphosphonates (Alendronate, Ibandronate, Risedronate, Zoledronic Acid)

Pros of Using Bisphosphonates (BPs) in Osteoporosis Treatment:

- Effective Fracture Prevention: BPs have been proven to significantly reduce the risk of fractures, particularly in the spine, hip, and wrist [134], by inhibiting osteoclast-mediated bone resorption. This effect helps maintain or improve bone density and structural integrity.
- Long-term Benefits: BPs have a long half-life in bone tissues, allowing for continued effects even after the cessation of therapy. This provides sustained benefits in reducing fracture risk over time [135].
- Versatile Dosing Options: BPs offer flexible dosing schedules, including daily, weekly, monthly, or even yearly infusions; especially the relatively longer intervals can improve patient adherence to treatment [136,137].
- Cost-effectiveness: many BPs are available in generic form, making them a cost-effective option for postmenopausal women [138,139].

Cons of Using Bisphosphonates (BPs) in Osteoporosis Treatment:

- Potential Side Effects: the use of BPs is associated with several potential side effects, such as gastrointestinal issues (e.g., esophagitis, gastric ulcers), acute phase reactions (fever, flu-like symptoms after infusions), and rare occurrences of osteonecrosis of the jaw and atypical femoral fractures [140,141].
- Long-term Safety Concerns: long-term use has raised concerns regarding the aforementioned rare complications, leading to recommendations for periodic evaluation of the necessity for continued therapy.
- Administration Requirements: oral BPs require specific conditions for administration, such as taking the medication with a full glass of water and remaining upright for at least 30 min to prevent esophageal irritation, which can be inconvenient for some patients [142].
- Renal Considerations: BPs are contraindicated in patients with severe renal impairment due to increased risk of toxicity, limiting their use in this population [143].

#### 14.1.2. Parathyroid Hormone (PTH) Analogs (Teriparatide, Abaloparatide)

PTH analogs, such as teriparatide and abaloparatide, promote new bone growth on both trabecular and cortical (periosteal and/or endosteal) surfaces by preferentially enhancing osteoblast activity rather than osteoclast activity. One double-blind RCT has demonstrated that abaloparatide significantly reduced the incidence of new vertebral and nonvertebral fractures compared with placebo and was associated with less frequent hypercalcemia than teriparatide, with rates of 3.4% and 6.4%, respectively [144]. Notably, it is better to detect any adverse effects of PTH analogs that might lead to early discontinuation. The rate of adverse events leading to early discontinuation was higher in participants receiving abaloparatide compared to those on placebo or teriparatide, mainly due to symptoms like nausea, dizziness, and palpitations [144]. The ACTIVE and ACTIVExtend trials were a phase 3, double-blind RCT and an amendment to ACTIVE, respectively [145,146], both of which showed that 18 months of abaloparatide treatment was well tolerated, significantly increased bone mineral density, and reduced fracture risks [147]. In this trial involving 1139 participants, adverse event rates were comparable between those previously treated with placebo and those treated with abaloparatide. Importantly, no instances of atypical femoral fractures (AFF) or osteonecrosis of the jaw (ONJ) were observed throughout the clinical trials [144,146]. However, the drugs in both studies were administered subcutaneously daily, which may be inconvenient or uncomfortable for some patients. Furthermore, the use of these drugs was generally limited to a 24-month period due to the potential risk of osteosarcoma observed in rat studies [148], although this risk has not been confirmed in humans.

#### 14.1.3. RANK-Ligand Inhibitor (Denosumab)

This drug is a fully human IgG2 monoclonal antibody that mimics the natural bone-protecting actions of OPG. It binds to and inhibits RANKL, a key factor involved in the formation and activation of osteoclasts, thus preventing bone loss. The biannual subcutaneous administration of 60 mg of denosumab offers a first-line OP treatment alternative for patients with low compliance to BPs because of gastrointestinal issues and renal impairment [149]. Moreover, administering denosumab subcutaneously twice yearly for 36 months has been shown to reduce the risk of vertebral, nonvertebral, and hip fractures in women with osteoporosis [150]. In terms of its long-term efficacy, in the pivotal 3-year FREEDOM trial, the relative risk of fracture in participants who were administered denosumab showed a reduction of 68%, 40%, 20%, and 16% for radiographic vertebral, hip, nonvertebral, and wrist fractures, respectively [150,151]. Additionally, results from the phase 3 randomized FREEDOM trial and open-label extension revealed that over a period of up to 10 years of denosumab treatment, there were sustained increases in BMD without reaching a plateau, a low incidence of fractures compared to initial trial results, and a low rate of adverse events (one AFF in each group with the extension period, seven cases of ONJ in the long-term group, and six cases of ONJ in the crossover group) [152].

#### 14.1.4. Sclerostin Inhibitor (Romosozumab)

Romosozumab (ROMO), a subcutaneously injected humanized monoclonal antibody, inhibits the protein sclerostin, thus enhancing Wnt signaling in osteoblasts to promote bone formation and reduce RANKL-mediated bone resorption by osteoclasts [153]. In postmenopausal women with osteoporosis at a high fracture risk, a 12-month treatment with ROMO followed by alendronate usage significantly reduced fracture risk compared to alendronate usage alone [154]. ROMO treatment for 6 and 12 months significantly improved BMD according to the real-world data of a prospective cohort study in Japan, which demonstrated that the percentage increase in BMD from baseline was 7.4% and 12.2% for the lumbar spine, 1.8% and 5.8% for the total hip, and 2.9% and 6.0% for the femoral neck, respectively. All changes were significantly greater than baseline (*p* < 0.001) [155]. Notably, 64 out of 230 patients in this trial experienced adverse events, the most frequent of which were injection site reactions associated with pain, swelling, and redness lasting 2 days or longer. By chance, one case of ONJ, one case of osteonecrosis of the leg, and one case of cerebral infarction were noted [155]. However, in light of pharmacovigilance analyses of ROMO reported mostly by U.S. and Japanese scholars, Japanese reports showed that patients were generally older and predominantly male compared to those from the U.S. Additionally, individuals with reported major adverse cardiovascular events tended to be older and more frequently used cardioprotective medications than those without cardiovascular events [156]. For the sake of patients’ health, it is essential to note that ROMO should not be used to treat patients who are at high risk for cardiovascular disease and stroke.

#### 14.1.5. Estrogen-Related Therapy (Tamoxifen, Raloxifene, Bazedoxifene Plus Estrogen)

In addition to traditional hormone replacement therapy directly employing estrogen and progesterone as hormone supplements, selective Estrogen Receptor Modulators (SERMs) prevent primary osteoporosis through their estrogen-like effects on bone tissues. They selectively bind to ERs on osteoblasts and osteoclasts, acting as partial E agonists to maintain bone density and reduce bone resorption. However, tamoxifen, the first generation of SERM, can serve as an antagonist in breast tissue but as an agonist in the uterus, thus increasing the risk of endometrial cancer [157]. Specifically, SERMs inhibit osteoclast activity to ameliorate bone breakdown while potentially stimulating osteoblasts to enhance bone formation. This dual action helps preserve or increase bone mass and strength, making SERMs particularly effective in managing osteoporosis in postmenopausal women [158].

Raloxifene, the second generation of SERM, was initially developed to antagonize ERs in breast tissue [159]. Nevertheless, the subsequent findings implicated that it not only functioned as an ER agonist in bone, but also generated protective effects in the context of endometrial cancer [157,159].

Bazedoxifene (BZD), the third generation of SERM, acts as an agonist on ERs in bone while exerting antagonistic effects on ERs in the breast and endometrium [159]. Particularly, BZD exhibits higher selectivity as a SERM compared to raloxifene, demonstrating a stronger antagonistic effect on the estrogen receptors in the endometrium [159]. Therefore, in 2013, the FDA approved a combination product of conjugated estrogens and bazedoxifene for treating moderate to severe menopausal vasomotor symptoms and for preventing postmenopausal osteoporosis in women [160,161]. Although SERMs show antiresorptive features in the treatment of osteoporosis, there were still several concerns regarding side effects. For instance, the most commonly reported side effects of raloxifene include hot flashes, flu-like symptoms, muscle cramps, arthralgia, and infections. Less frequent side effects may include insomnia, nausea or vomiting, sinusitis, deep vein thrombosis (DVT), bronchitis, pharyngitis, and breast pain [162]. According to the Osteoporosis Treatment Clinical Trial (MORE) of raloxifene, the safety of raloxifene in the treatment of osteoporosis was assessed in a large (7705 patients), multinational, placebo-controlled trial revealing higher rates of peripheral edema (14.1% for raloxifene vs. 11.7% for placebo), muscle spasms or leg cramps (12.1% vs. 8.3%), hot flashes (7.8% vs. 4.7%), cholelithiasis (3.3% vs. 2.6%), and venous thromboembolic events (2.0% vs. 1.4%) [163].

#### 14.1.6. Calcitonin Salmon

Calcitonin salmon (sCT), a synthetic form of the hormone calcitonin derived from salmon, has been used in the treatment of osteoporosis. Its mechanism of action is primarily focused on inhibiting bone resorption [164].

In 2000, one randomized trial reported that nasal spray containing sCT at a dose of 200 IU daily significantly lowered the risk of new vertebral fractures in postmenopausal women with osteoporosis [165]. However, nearly 20 years later, in 2021, a meta-analysis conducted by Ning Li et al. concluded that intranasal sCT did not clearly enhance bone mineral density in the lumbar and hip areas, which might be explained by the variations of measurement techniques, methods, and chosen subjects among the different studies [166]. Nevertheless, this meta-analysis still showed that intranasal sCT outperformed conventional medications in lowering blood calcium levels, elevating the creatinine ratio and increasing alkaline phosphatase.

Additionally, a phase 3 clinical trial demonstrated that oral recombinant sCT was more effective than nasal synthetic sCT and placebo in enhancing BMD and decreasing bone turnover, which may be an alternative treatment option for postmenopausal osteoporosis [167].

Generally, calcitonin salmon has mild and infrequent adverse effects [168], although serious hypersensitivity reactions, such as bronchospasm, throat swelling, anaphylactic shock, and death due to anaphylaxis, have been reported [169]. Such adverse effects are more frequent with injections than with nasal spray. For parenteral administration, gastrointestinal issues like transient nausea, sometimes accompanied by vomiting, are common (1 out of 10) and can be mitigated by administering the drug at bedtime [170]. Other GI symptoms include poor appetite, abdominal discomfort, and a metallic taste. Nasal spray patients report fewer GI symptoms, with nausea occurring in only 1.8% of cases [170].

### 14.2. Non-Pharmacological Treatments

#### 14.2.1. Nutritional Support

Although medication is often the primary choice for reducing osteoporosis and subsequent fracture risk in the elderly, it may not always be practical. Instead, dietary adjustments, such as increasing the intake of calcium, vitamin D, and protein, can be a more viable option [171]. Also, it is important to take other vitamins and trace elements such as magnesium into consideration to prevent osteoporosis.

Dietary proteins can influence the secretion and activity of insulin-like growth factor I (IGF-I), which is a critical orthotropic hormone for bone formation [172]. Moreover, dairy products (milk, cheese, and yogurt) are key dietary sources of bone-supporting nutrients like calcium, phosphorus, and magnesium, all of which play crucial structural roles in maintaining healthy bones.

Vitamin K is responsible for the post-translational conversion of glutamyl to -carboxyglutamyl residues in osteocalcin to facilitate the mineralization process for the purpose of osteoporosis prevention [173,174]. Vitamin C, indicative of a healthy diet abundant in fruits and vegetables, can lead to the suppression of osteoclast activity due to its antioxidant properties [175]. In 2018, a meta-analysis conducted by Sun et al. robustly supported the idea that boosting dietary vitamin C intake could reduce the risk of hip fractures in both men and women [176].

Magnesium (Mg) deficiency directly influences osteoporosis by means of affecting crystal formation and bone cell activity [177]. Furthermore, the evidence shows that women with low bone density often have reduced serum levels of magnesium and albumin, as well as low levels of calcium and trace minerals [178,179]. One meta-analysis conducted by Jinlei Chang et al. has also revealed that postmenopausal women suffering from osteoporosis tend to have reduced serum Mg levels. Therefore, higher Mg supplementation is necessary to lower future osteoporosis risk [180]. However, there are still concerns about supplementing the general population with magnesium, as excessive amounts of the mineral may adversely affect bone health [181].

#### 14.2.2. Exercise and Its Impact on Osteoporosis

Physical activity is recommended for OP prevention based on evidence that it helps maintain bone health, stimulates bone formation and mineral accumulation, strengthens muscles, improves balance, and consequently reduces the overall risk of falls and fractures. For instance, a meta-analysis of Tai Chi (TC) practice to prevent the risk of fall conducted by Lomas-Vega et al. has revealed that practicing TC may decrease the incidence of falls resulting in injury by about 43% and 50%, respectively, over a short term period (<12 months), which can improve postural stability [182]. A systematic review by Marina B. et al. has also demonstrated that physical activity likely contributes to the prevention of OP, with stronger evidence supporting its impact on lumbar spine BMD compared to hip BMD [183]. Some studies further indicate that short-term step aerobics exercise, resistance training, or whole-body vibration (WBV) training help maintain or increase bone mass and BMD in postmenopausal women, thus improving their health and quality of life [184,185,186].

Interestingly, from the molecular point of view, exercise mitigates the detrimental changes associated with OP by influencing apoptosis, inflammatory responses, and autophagy. Additionally, it may impact the epigenetic mechanisms of bone metabolism through the regulation of non-coding RNAs and DNA methylation [187].

To sum up, it is a global trend that exercise can lead to a healthy lifestyle and helps prevent OP, as opposed to a sedentary life. By incorporating regular physical activity into daily routines, individuals not only strengthen their bones but also enhance overall well-being and reduce the risk of chronic diseases. This proactive approach is essential for maintaining bone health and functional mobility, particularly as one ages.

#### 14.2.3. Antioxidants and Natural Products

The upcoming discussion will mainly summarize recent findings on how reactive oxygen species (ROS) can influence osteoporosis. Moreover, this paragraph will highlight classic examples of how antioxidants and natural products can help alleviate and prevent osteoporosis.

Oxidative stress (OS) causes cellular damage through the oxidation of lipids, alteration of membrane structures, and oxidation of proteins and nucleic acids. This damage can affect entire organs and potentially lead to systemic issues [188]. One of the most representative diseases is osteoporosis, especially osteoporosis in postmenopausal women and elderly men [189]. In the former, it is related to OS secondary to estrogen deficiency, which induces the activation of NADPH oxidase and/or a reduction in the synthesis of antioxidant enzymes and glutathione (GSH) levels [189,190,191]. In the latter, there is more emphasis on aging, which typically involves an excessive buildup of ROS and oxidative stress, which promote osteoclast activity and subsequent bone loss [192].

It is intriguing to discover different kinds of antioxidants and natural products among fruits, vegetables, herbs, and woody plants. Polyphenols and anthocyanins are the most prevalent antioxidants in our diet, commonly found in our daily life.

#### 14.2.4. Green Tea Catechins

As one of the main types of catechins, epigallocatechin gallate (EGCG) possesses the most potent antioxidant and free radical scavenging abilities [193]. Several studies have also shown that these beneficial effects of green tea extracts (GTE) can reduce OS [194,195]. In 2020, a comprehensive systemic review performed by Huang et al. demonstrated that catechins can boost osteoblastogenesis by promoting the osteogenic differentiation of mesenchymal stem cells (MSCs) and enhancing the survival, proliferation, differentiation, and mineralization of osteoblasts [196]. The positive effects of catechins on osteogenesis, observed in vitro, have been validated through various murine studies [197,198,199] and corroborated by epidemiological observations in human subjects [200,201]. Intriguingly, two human clinical trials conducted by Shen et al. demonstrated, firstly, that GTE supplementation, Tai Chi exercise, and their combination each enhanced muscle strength in postmenopausal women suffering from osteopenia during a 6-month period [201]. The second study regarding GTE dosing and Tai-Chi exercise demonstrated a safety profile for liver and kidney function, along with improved scores for role-emotional and mental health of subjects like postmenopausal women with osteopenia. However, there was no impact on quality of life [202].

#### 14.2.5. Lycopene

Considering the plentiful presence of lycopene in human food like guavas, watermelon, cooked tomatoes, papaya, grapefruit, and mangos, along with its exceptional antioxidant capabilities, it is fascinating to investigate the role of lycopene in preventing osteoporosis. For instance, limiting the intake of dietary lycopene for one month significantly elevates biomarkers of oxidative stress and bone resorption in postmenopausal women [203]. Furthermore, lycopene appears to promote an anabolic state in bone metabolism, thus enhancing bone tissue health by stimulating osteoblastogenesis and inhibiting osteoclastogenesis, which was observed in a study focusing on the human osteoclast and osteoblast precursor cells treated with 500 nM lycopene for 21 days [204]. A randomized controlled trial carried out by Mackinnon et al. showed that lycopene supplementation in postmenopausal women significantly boosted antioxidant capacity while reducing oxidative stress and the bone resorption marker N-telopeptide (NTx), suggesting that lycopene may lower bone resorption markers and potentially reduce the risk of osteoporosis [205].

#### 14.2.6. Resveratrol

Resveratrol (RSV), a naturally occurring polyphenolic compound found primarily in grapes, red wine, and certain berries (blueberry, cranberry, mulberry), has garnered considerable interest for its potential bone benefits.

One experimental rat study utilizing an ovariectomized model demonstrated that compared to the surgery placebo group, the ovariectomized group showed significant reductions in bone calcium content and BMD (*p* < 0.05), while resveratrol mitigated and reversed the condition dose-dependently. In osteoblasts of the ovariectomized rats, the levels of VEGF and COL1A1 were significantly reduced, while RANKL increased in osteoclasts. Resveratrol reversed these effects in a dose-dependent manner [206]. Relatively convincingly, a 24-month, double-blind, randomized, placebo-controlled trial proved that regular supplementation with 75 mg of resveratrol twice daily could potentially slow bone loss in the lumbar spine and femoral neck, which was accompanied by a 7.24% decrease in the levels of C-terminal telopeptide type-1 collagen, a marker of bone resorption, compared to placebo [207].

## 15. Discussion

Osteoporosis, often described as a “silent disease,” predominantly affects postmenopausal women but also significantly impacts older men, particularly in terms of fracture-related mortality. Recognizing the gender-specific nuances of osteoporosis is crucial for developing comprehensive public health strategies and clinical practices.

Osteoporosis manifests differently in men and women due to variations in hormonal regulation, receptor status, and bone physiology. Women experience a rapid decline in bone mass post-menopause because of a sharp drop in estrogen levels, which leads to increased bone resorption. Men, on the other hand, experience a more gradual bone loss associated with a steady decline in testosterone levels with age. Understanding the differences between them is vital for developing gender-specific prevention and treatment strategies.

Sex hormones play a crucial role in bone remodeling, a process involving the resorption of old bone by osteoclasts and the formation of new bone by osteoblasts. Estrogen and testosterone are key regulators of this process, influencing the activity and lifespan of these bone cells.

Estrogen deficiency, particularly post-menopause, accelerates bone resorption by increasing osteoclast activity. This pathway is mediated through a rise in pro-inflammatory cytokines (e.g., IL-1, IL-6, TNF-α) and RANKL, which promote osteoclastogenesis. Estrogen inhibits osteoclast formation by promoting the production of osteoprotegerin (OPG), a decoy receptor for RANKL, thereby preventing RANKL from binding to its receptor RANK on osteoclasts. Furthermore, estrogen promotes osteoblast survival and function via activation of the Wnt signaling pathway. The decrease in estrogen levels post-menopause reduces the production of OPG, leading to enhanced RANKL activity and increased bone resorption.

Likewise, androgens play a critical role in bone metabolism, primarily through their conversion to estrogen in men. Via this conversion, testosterone promotes osteoblast proliferation and differentiation while inhibiting osteoclast formation. In aging men, decreased testosterone levels, exacerbated by conditions like androgen deprivation therapy (ADT) for treating prostate cancer, lead to lower BMD and deteriorated bone microarchitecture. Understanding the direct and indirect actions of testosterone on bone cells highlights the importance of maintaining adequate androgen levels for bone health in aging men.

To sum up, the onset of osteoporosis is closely linked to hormone deficiency, which disrupts the balance of bone remodeling. In postmenopausal women, the sharp decline in estrogen levels results in increased bone resorption and decreased bone formation, leading to rapid bone loss. In men, the gradual decline in testosterone levels leads to a slower but steadier loss of bone mass. Conditions such as hypogonadism and the use of ADT in prostate cancer patients further exacerbate bone loss and increase fracture risk. Understanding the mechanisms through which hormones influence bone cells is important for developing targeted therapies.

Androgen can be defined as a high affinity ligand for the androgen receptor (AR). Also known as NR3C4 (nuclear receptor subfamily 3, group C, gene 4), the AR is part of the steroid hormone nuclear receptor family, and serves as a ligand-inducible transcription factor. This group of the steroid hormone nuclear receptor family also includes the estrogen receptor (ER), glucocorticoid receptor (GR), progesterone receptor (PR), and mineralocorticoid receptor (MR). Similar to other members of the nuclear receptor family, the AR is composed of three main functional domains: the N-terminal domain (NTD), followed by the DNA binding domain (DBD), and concluding with the C-terminal ligand binding domain (LBD). In the absence of androgen, AR is found in the cytoplasm, where it is bound to heat shock proteins. Acting from the cytoplasm to the nucleus, androgen first binds to the AR in the cytoplasm, and then the forming A/AR complex is translocated into the nucleus by importin-α. Within the nucleus, the AR identifies and attaches to the corresponding genomic areas that contain sequence motifs known as androgen response elements (AREs), functioning as a dimer. The majority of AR binding sites (ARBSs) are located in the promoter and enhancer regions of target genes. Under the androgen receptor knockout male mice (ARKO) model, mechanically, Chang and Kang et al. [95] pinpointed several key genes that are essential for AR-mediated bone formation, including the tissue-nonspecific alkaline phosphatase (TNSALP)-encoded alkaline phosphatase 2 (Akp2) gene and the small integrin-binding ligand N-linked glycoprotein (SIBLING) gene family. Acting as an intermediate product of the special pathway, the A/AR complex can stimulate the expression of the TNSALP and SIBLING gene family by attaching to the ARE motifs located in their promoter region.

Estrogen exerts its effects by combining with the estrogen receptors ERα, ERβ, and G-protein coupled estrogen receptor 1 (GPER1). In the classic genomic pathway, estrogen binds to ERs in the cytoplasm, forming an E/ER complex that migrates to the nucleus and binds to estrogen response elements (EREs) on DNA, regulating gene transcription. This process is crucial for maintaining bone density by promoting osteoblast activity and inhibiting osteoclastogenesis. In contrast, the non-genomic pathway involves rapid signaling cascades initiated at the cell membrane, influencing cellular functions without directly altering gene expression. These pathways (genomic and non-genomic) are important targets for developing drugs that mimic estrogen’s protective effects on bone but act without the estrogen-associated risks.

Given the complicated interplay between sex hormones and bone metabolism, future research should focus on developing targeted therapies that address the specific needs of both men and women. Hormone replacement therapy (HRT) has shown promise but comes with associated risks and side effects. Thus, searching for safer and more effective alternatives is crucial. Developing selective estrogen receptor modulators (SERMs), selective androgen receptor modulators (SARMs), and other hormone analogs can offer safer alternatives to traditional HRT. For instance, bazedoxifene combined with conjugated estrogens has shown efficacy in reducing fracture risk without significantly increasing cancer risk.

Research into both genomic and non-genomic pathways of sex steroid action on bone cells can uncover new therapeutic targets. Ligand-independent actions and rapid signaling pathways of estrogen and androgen receptors can be harnessed to develop new drugs that provide bone protection without the side effects of traditional hormone therapies. The role of GPER1 in estrogen signaling, for example, offers a novel pathway for therapeutic intervention.

Personalized treatment plans that consider hormonal status, genetic predisposition, and individual risk factors can enhance treatment outcomes. For men with an androgen deficiency, testosterone replacement therapy (TRT) could be beneficial, although further studies are needed to confirm its long-term safety and efficacy.

Lifestyle modifications, including diet and exercise, are essential components of osteoporosis management. Nutritional support with adequate calcium, vitamin D, and other essential nutrients, alongside regular weight-bearing and resistance exercises, can improve bone density and strength. Exercise promotes mechanical loading on bones, which stimulates osteoblast activity and bone formation.

Antioxidants and natural products might mitigate the effects of oxidative stress on bone health. Although evidence suggests that antioxidants like GTE, lycopene, and RSV may show effectiveness in supporting bone health to fight against osteoporosis and reduce oxidative stress, it is crucial to note that individual responses can vary due to differences in metabolic predisposition. Additionally, the dosage of these antioxidants can differ with each intake, which could affect their efficacy. Therefore, it is vital to pay close attention to these factors when considering antioxidant supplements for osteoporosis prevention.

The current study has reviewed the bone remodeling, hormone actions, hormone receptor status, and therapeutic targets of primary osteoporosis. However, many detailed cellular and molecular mechanisms underlying primary osteoporosis seem complicated and unexplored, and warrant further investigation.

## 16. Conclusions and Future Perspectives

The pathophysiology of primary osteoporosis induced by androgen and estrogen deficiency involves complex hormonal interactions affecting bone metabolism. Its effective management requires early diagnosis, personalized treatment strategies, lifestyle modifications, and the exploration of novel therapeutics. Continued research into the molecular mechanisms and potential benefits of antioxidants and natural products may further enhance treatment options, ultimately improving the quality of life for individuals affected by osteoporosis. Additionally, future research should focus on developing innovative therapeutics, including SERMs, SARMs, and other hormone analogs, to provide safer and more effective alternatives to traditional HRT. Exploring both genomic and non-genomic pathways of sex steroid action on bone cells can uncover new therapeutic targets, leading to drugs that offer bone protection without the significant adverse effects associated with conventional hormone therapies. By addressing gender-specific aspects and exploring innovative treatment avenues, we may develop more effective and safer strategies to withstand this debilitating disease in the near future.

## Figures and Tables

**Table 1:** Modifiable and non-modifiable risk factors of osteoporosis.

Modifiable Risk Factors	Description
Smoking	Nearly doubles the risk of hip fracture by reducing bone density and vascular health.
Excessive alcohol intake	More than 2 units daily increases fracture risk by weakening bone structure.
Low body mass index	BMI below 19 is linked to decreased bone mass and higher fracture risk.
Poor nutrition	Low intake of calcium, protein, fruits, and vegetables compromises bone health, especially in older adults.
Low dietary calcium intake	Insufficient calcium intake, worsened by age-related absorption decline, weakens bones.
Vitamin D deficiency	Common in older adults with limited sun exposure, leading to poor calcium absorption and lower bone density.
Inactivity (not enough exercise)	Physical inactivity accelerates bone loss and muscle weakening, increasing fracture risk.
Eating disorders	Disorders like anorexia lead to weight loss, estrogen deficiency, and rapid bone loss, especially in young women.
Frequent falls	Frequent falls heighten fracture risk in those with compromised bone health.
**Non-Modifiable Risk Factors**	**Description**
Age	Advanced age is strongly linked to osteoporosis, with most fractures occurring in those over 65.
Gender	Women have a higher risk due to hormonal factors and lower peak bone mass.
Ethnicity	Individuals of Caucasian and Asian descent have a higher risk of developing osteoporosis compared to those of African or Hispanic descent.
Family history of osteoporosis	A family history increases susceptibility due to genetic and lifestyle influences.
Body frame size	Individuals with smaller skeletal frames have a lower baseline bone mass, increasing their vulnerability to osteoporosis and fractures as they age.

**Table 2:** Possible medical conditions or treatments that can induce secondary osteoporosis.

Contributors	Examples/Conditions
**Lifestyle Changes**	Alcohol abuse, excessive thinness, excess vitamin a, frequent falling, high salt intake, immobilization, inadequate physical activity, low calcium intake, smoking (active or passive), vitamin D insufficiency/deficiency
**Genetic Diseases**	Cystic fibrosis, Ehlers-Danlos, Gaucher’s disease, hemochromatosis, hypophosphatasia, hypophosphatemia, Marfan syndrome, Menkes steely hair syndrome, osteogenesis imperfecta, parental history of hip fracture, porphyria, homocystinuria
**Gastrointestinal Disorders**	Celiac disease, gastric bypass, gastrointestinal surgery, malabsorption, inflammatory bowel disease, pancreatic disease, primary biliary cirrhosis
**Thyroxotoxicosis**	(Specific examples were not listed, but this would include any condition leading to excess thyroid hormone.)
**Hematologic Disorders**	Hemophilia, leukemia and lymphomas, sickle cell disease, multiple myeloma, monoclonal gammopathies, systemic mastocytosis, thalassemia
**Endocrine Disorders**	Obesity, Cushing’s syndrome, diabetes mellitus (types 1 and 2), hyperparathyroidism
**Hypogonadal States**	Anorexia nervosa, androgen insensitivity, female athlete triad, hyperprolactinemia, hypogonadism, panhypopituitarism, premature menopause (<40 years), Turner’s and Klinefelter’s syndromes
**Rheumatologic and Autoimmune Diseases**	Ankylosing spondylitis, other rheumatic and autoimmune diseases, rheumatoid arthritis, systemic lupus
**Neurological and Musculoskeletal Factors**	Epilepsy, muscular dystrophy, multiple sclerosis, Parkinson’s disease, spinal cord injury, stroke, proximal myopathy
**Other Conditions and Diseases**	AIDS/HIV, amyloidosis, chronic obstructive lung disease, congestive heart failure, chronic metabolic acidosis, depression, renal disease, hypercalciuria, idiopathic scoliosis, post-transplant bone disease, sarcoidosis, weight loss
**Medications**	Aluminum-containing antacids, androgen deprivation therapy, anticoagulants (unfractionated heparin), anticonvulsants (e.g., phenobarbital, phenytoin, valproate), aromatase inhibitors, barbiturates, cancer chemotherapeutic drugs, Cyclosporine A and tacrolimus, glucocorticoids (≥5.0 mg/day prednisone or equivalent for ≥3 months), gonadotropin releasing hormone agonists and antagonists, Depot medroxyprogesterone acetate (Depo-provera), Methotrexate, parenteral nutrition, proton pump inhibitors, selective serotonin reuptake inhibitors, Tamoxifen (premenopausal use for breast cancer treatment), thiazolidinediones (such as pioglitazone and rosiglitazone), thyroid replacement hormone (in excess)

**Table 3:** A summary of impacts on bone in different diseases associated with androgen deficiency.

Astrogen Deficiency	Clinical Manifestation	Impacts on Bone in Adults or Teenager
Androgen insensitivity syndrome (AIS)	- Complete AIS with a normal external female phenotype in girls and women under 46,XY - At puberty, normal breast development and typical female distribution of fat - Depends on residual AR with different levels of undermasculinization	Decreased BMD in the region of lumbar and femoral neck in congenital AIS
Cryptorchidism	Decreased testicular hormone production later in life Ectopic testis, outside the normal path of testicular descent	One case report of 65 year-old male presenting with multiple thoracic vertebral fractures, and reduced vertebral height Could not reach a normal pubertal peak bone level in both adolescent and adult
Klinefelter’s syndrome (KS)	Taller than average stature, longer legs, shorter torso and broader hips compared with other boys. Absent, delayed or incomplete puberty. After puberty, less muscle and less facial and body hair compared with other teens. Small, firm testicles and small penis. Enlarged breast tissue (gynecomastia)	Decreased in bone mass in 25–48% of KS cases A deficiency associated with a significant decrease in BMD leads to the risk for premature osteopenia and osteoporosis A meta-analysis of testosterone replacement therapy in KS patients confirmed its limited impact on bone health, showing a significant increase in lumbar BMD but not in femoral neck BMD.
Orchiectomy for Prostate Cancer	Patients with prostate malignancy Low sex drive and depression Low muscle mass	With bilateral orchiectomy, the rate of loss in BMD is estimated at approximately 8% to 10% over the first 1 to 2 years BMD was measured over 6–42 months, showing an average hip BMD decrease of 7.6% following the first orchiectomy.
Androgen deprivation therapy (ADT)	Patients with prostate malignancy - ADT side effects, such as breast enlargement, mastodynia, hot flashes, altered fat distribution - Reduced testicular volume	■ A significant decrease in BMD for the hip, spine, whole body, and upper limbs (*p* < 0.001) ■ A 5–10 fold greater bone density loss at multiple skeletal sites compared to healthy controls or prostate cancer patients not on ADT ■ Bone loss is greatest in the first year of ADT, indicating the need for early preventive therapy

**Table 4:** A summary of impacts on bone in different diseases associated with estrogen deficiency.

Estrogen Deficiency	Clinical Manifestation	Impacts on Bone in Adults or Teenager
Turner syndrome (TS)	- Short stature, short and webbed neck - “shield” chest with the appearance of widely spaced nipples - Congenital lymphedema of the hands and feet	■ Abnormal trabecular microarchitecture and lower cortical bone porosity of the radius and tibia ■ The risk of fractures two times higher than general population, especially at metacarpal bones, femoral neck, lower spine, and forearm
Primary ovarian insufficiency (POI)	Cessation of ovarian function before the age of 40 years Early vasomotor symptoms, urogenital atrophy Significantly higher triglyceride (TG) levels and lower high-density lipoprotein cholesterol (HDL-C) levels compared to controls	Spinal BMD and femoral neck BMD were significantly lower in POI patients than in healthy controls Young women who experience ovarian dysfunction before reaching their peak adult bone mass
Anorexia Nervosa (AN)	Restriction of energy intake that leads to a low body weight and reduced hormone source Intense fear of gaining weight or becoming fat, persistent behavior that prevents weight gain, despite being underweight Distorted perception of body weight and shape	Both cortical and trabecular bone decreased, leading to significant loss of bone mass Increased marrow adipose tissue and decreased bone integrity are associated with low BMD
Aromatase deficiency (AD)	Mutations in the *CYP19A1* gene that leads to ambiguous genitalia in 46,XX fetuses, who do not develop secondary sexual characteristics Progressive virilization like severe acne, lowered voice, hirsutism and clitoromegaly	Continued linear bone growth in female and retarded bone age in puberty (girls) Genu valgum in male and retarded bone age with unfused epiphyses
Autoimmune oophoritis (AO)	The presence of enlarged cystic ovaries, elevated anti-adrenal antibodies (and often primary adrenal insufficiency), evidence of theca cell (but not granulosa cell) destruction and elevated anti-oocyte antibodies Symptoms similar to POI	Immune disruption contributes to pathological bone loss via the influence of inflammatory markers and autoantibodies. The relation of autoimmunity disease and decreased bone mass density seemed high Lower BMD at L1–L4, femoral neck, and total hip
Female pure gonadal dysgenesis (46,XX)	Congenital adrenal hyperplasia caused by mutations in the gene coding for 21-hydroxylase Usually present with primary amenorrhea and absence of secondary sexual characteristics	Similar to TS, a marked decrease in BMD of the lumbar spine and femoral neck
Constitutional delay of growth and puberty	A self-limited condition in which puberty starts later than usual but progresses normally, more commonly encountered in boys than in girls Short term use of estradiol promotes estrogenic pubertal signs and may induce bone maturation	Delayed bone growth but not stalled and delayed bone age compared with peers Hormone replacement therapy has been shown to improve the bone density in puberty
